# Acquired Iris Heterochromia After Pars Plana Vitrectomy

**DOI:** 10.7759/cureus.24234

**Published:** 2022-04-18

**Authors:** Ayat Haroun, Saif Aldeen AlRyalat, Maen Abdallah, Mutaz Hararah, Almutez Gharaibeh

**Affiliations:** 1 Department of Ophthalmology, The University of Jordan, Amman, JOR; 2 Department of Ophthalmology, Eye Speciality Hospital, Amman, JOR

**Keywords:** iris, ophthalmology, pars plana vitrectomy, heterochromia iridum, acquired

## Abstract

Describe a case of acquired heterochromia after intraocular surgery. A 63-year-old healthy female patient presented to the eye clinic with rhegmatogenous retinal detachment in her left eye. She underwent uncomplicated pars plana vitrectomy with implantation of posterior chamber intraocular lens. One week after the surgery the patient noticed a change in the color of her operated eye (green instead of blue), she came back to the clinic complaining about her eye color, weeks later her eye color returned back to blue. This case shows a unique presentation of transient acquired heterochromia after intraocular surgery in an adult patient and emphasizes the importance of counseling and reassuring patients regarding the possibility of this event.

## Introduction

Heterochromia irides are defined as an asymmetrical iris color in one eye in relation to the other, which could be complete or sectoral involving one part of the iris [1]. This condition has been described centuries before, it was mentioned that the eastern emperor Anastasio 1 was called Dicorus because his eyes were of different colors [2]. Heterochromia could be congenital or acquired. Congenital cases could be isolated and benign but also can be associated with syndromes, such as Sturge-Weber syndrome, Waardenburg syndrome, and Horner syndrome. Acquired cases could be due to several causes, including melanocytic proliferation in melanoma, diffuse iris nevus, impaired sympathetic tone, sympathetic chain tumors, intraocular inflammation, ocular trauma, intraocular foreign body, and after certain eye drops like prostaglandin analogs [3].

## Case presentation

We report a case of a previously healthy 63-year-old female, who presented to the eye clinic with a sudden painless drop of the left eye (OS) central vision progressively worsening over the past 4 days. The ophthalmic examination showed corrected distance visual acuity Log MAR 0.0 (6/6, 20/20, 1.0) in the right eye (OD), and hand motion in the left eye. Pupils were round, regular, and reactive in the right eye, whereas a relative afferent pupillary defect was noticed in the left eye. The anterior segment was examined to be unremarkable in both eyes. Posterior segment examination showed dense vitreous hemorrhage obscuring the fundus view in the left eye; B-scan ultrasound was performed, and rhegmatogenous retinal detachment with dense vitreous hemorrhage was observed, OD fundus was examined to be normal. The patient was planned for combined left pars plana vitrectomy and cataract surgery. The surgeon decided to do cataract surgery since the patient had a moderate nuclear sclerosis cataract which is very likely to progress after vitrectomy. The surgery was done under conscious sedation starting with uneventful phacoemulsification with implantation of a one-piece acrylic intraocular lens in the capsular bag, 23-gauge pars plana vitrectomy was done, core vitrectomy with removal of vitreous hemorrhage, on careful inspection, there was a superior detachment involving the macula and extending 4 clock hours with a horseshoe break and no proliferative vitreoretinopathy (PVR) and no subretinal hemorrhage. Perfluorocarbon was used to flatten the retina, endolaser photocoagulation was carried out around the break, and no tamponading agent was left in the eye given the superior location of the break and the absence of PVR, the overall operative time was 85 minutes and at the conclusion of the surgery subconjunctival antibiotics and steroids were given.

Postoperative day one exam: visual acuity was counting fingers 3 m in the left operated eye. Anterior segment examination showed a dense inflammatory reaction +4 cells with +3 flare but no hyphema, no fibrinous material seen, and iris color was still blue and symmetrical. Posterior segment examination showed a flat retina. The patient was started on prednisolone acetate 1% eye drops every 1 h, ofloxacin 0.3% eye drops four times a day, tobramycin eye ointment at bedtime, and cyclopentolate eye drops at bedtime. She was then scheduled for the next follow-up visit.

**Figure 1:**
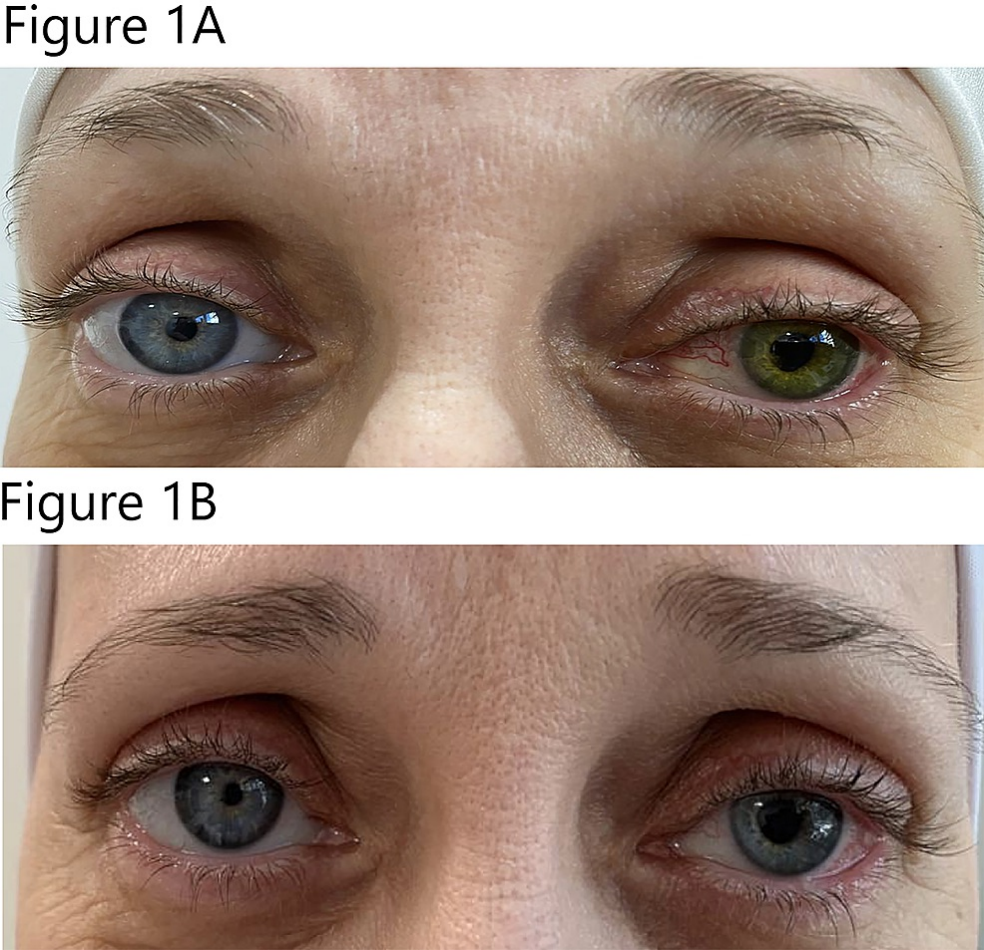
Colored photograph of both eyes. One week after surgery (1A), and three weeks after surgery (1B).

One week after the surgery, the patient noticed a patchy darkening of her operated eye, upon examination visual acuity was counting fingers 3 m, the anterior chamber inflammation was examined to be settling down, the retina was flat as well as the choroid on the B-scan done one-week postoperatively, she complained about the change in her eye color, which she believed was due to the operation (Figure 1A). We then told her that the operation was performed to repair the detached retina and this was an unexpected event. The regimen of postoperative treatment was continued as the following, topical antibiotics and cycloplegics with the same frequency continued for another week and then stopped while topical steroid was tapered slowly over 4 weeks. Three weeks later, her eye color returned to its original one, and her best-corrected visual acuity was Log MAR 0.7 (6\30,20\100,0.2) (Figure 1B). The patient gave us permission to use her photos.

## Discussion

Eye color differs among humans, which ranges from light blue to dark brown. It is determined by several genes as was demonstrated by White and Smith, who found out that the development of eye color is controlled by 16 different genes playing together in a complex manner, with OCA2 and HERC2 genes being the two main players [4,5]. Although the number of melanocytes is the same in blue and dark eyes [6], eye color also varies according to morphological factors such as the pigment granules of the posterior pigment epithelium, their concentration and type within the stromal melanocytes, the light scattering and absorption properties of the extracellular stromal matrix [7,8]. Sympathetic nervous system proper development also plays a role. Despite the migration of melanocytes from the neural crest into the iris being preprogrammed, acquired heterochromia in adults with acquired sympathetic denervation as in Horner syndrome could be attributed to local trophic factors influencing the distribution and amount of pigmentation in these eyes [9]. This was also suggested by Imesch et al., who reported that the number of stromal melanocytes and their tyrosinase activity may be regulated by sympathetic innervation even beyond childhood [1].

In terms of studying iatrogenic etiologies in heterochromia, there are some cases mentioned in the literature. Acquired heterochromia had been noticed in infants after a congenital cataract operation. A study conducted by Lenart et al. showed that the darkening of iris color in the operated eyes of children with congenital cataract in relation to the non-operated eye could be due to massive prostaglandin release stimulated by the cataract surgery that may occur through the same mechanism through which latanoprost causes the darkening of iris color [10]. Prostaglandins mediate their effect on iris color by affecting the response of melanocytes to neuronal stimuli, increasing melanocytes proliferation and tyrosinase activity, and decreasing the breakdown of melanosomes [1]. This effect of prostaglandin is seen to be variable based on the original eye color itself [11].

Others had noticed transient iris heterochromia after glaucoma drainage device implantation, which was done on both eyes; however, only one eye demonstrated iris heterochromia, and they said it could be due to the serous-hemorrhagic choroidal effusion they found postoperatively with continuous iron deposition even if blood cells in the anterior chamber or the vitreous cavity had never been found during ophthalmological examination [12], our patient had attached retina postoperatively with no choroidal effusion evident on B-scan ultrasonography.

In another case report, iris heterochromia was seen after the use of tissue plasminogen activator (T-PA) and sulfur hexafluoride (SF6) gas injection, it was speculated to be either because of the toxic effect from the T-PA used or it may have been a consequence of the persistent bleeding with iron deposition on the iris [13]. Our patient did not have any tamponing agent nor T-PA injection.

Ebola virus associated with panuveitis and heterochromia was seen in another case report in which the patient eye color changed unilaterally from blue to green. This effect coincided with the excessive inflammatory response seen in the iris and ciliary body as demonstrated via anterior segment optical coherence tomography (OCT) and ultrasound biomicroscopy with both showing edema and thickening; the reversal of this heterochromia after the inflammation subsided supported the suggestion that excessive leukocyte inﬁltration could be the cause [14].

In this case, there was no breaching of the posterior capsule during the operation; however, we cannot exclude the fact that the microscopic leak of red blood cells or their metabolic product can be a contributor to the transient heterochromia observed. Moreover, increased iris pigmentation with the resulting temporary intense postoperative inflammatory response might play a role in this transient observation.

## Conclusions

Acquired iris heterochromia can result from several causes, including intraocular surgery such as pars plana vitrectomy and cataract surgery. In this study, we report a case of transient acquired iris heterochromia after combined phaco and pars plana vitrectomy in an adult patient. although the rarity of this condition, The results suggest that surgeons should be aware of the possibility of this event in order to counsel patients properly and reassure them.
